# A Case-Based Approach to the Management of Corneal Melts and Perforations in Ocular Surface Disorders

**DOI:** 10.7759/cureus.81293

**Published:** 2025-03-27

**Authors:** Abha Gour, Aastha Garg, Kajal Singh, Nikunj V Patel, Virender S Sangwan

**Affiliations:** 1 Department of Cornea, Dr. Shroff's Charity Eye Hospital, Delhi, IND; 2 Department of Cornea and Anterior Segment, Dr. Shroff's Charity Eye Hospital, Delhi, IND

**Keywords:** chronic inflammation, corneal perforation, limbal stem cell deficiency (lscd), ocular surface disorders, visual rehabilitation

## Abstract

Corneal perforations caused by chronic ocular surface disorders present significant management challenges and can lead to blindness if untreated. This case series reviews the pathophysiology of corneal melts and examines treatment strategies tailored to the size, location, and etiology of perforations in conditions such as Stevens-Johnson syndrome, graft-versus-host disease, and chemical injuries. Through detailed case analyses, various interventions, including cyanoacrylate glue, Tenon’s patch grafting, mucous membrane grafts, scleral patch grafts, and conjunctival flaps, were evaluated, along with emerging therapies like biosynthetic hydrogels and collagen-like peptides. Findings highlight the role of chronic inflammation and adnexal abnormalities in disrupting ocular surface integrity, affecting treatment outcomes. This case series underscores the importance of a comprehensive approach that not only repairs corneal defects but also addresses underlying systemic and ocular conditions, with advancements in biosynthetic materials showing promise for improving patient outcomes.

## Introduction

Ocular surface inflammatory disorders arise from direct injury or systemic conditions that trigger persistent ocular adnexal connective tissue inflammation. These disorders can be broadly classified into acute and chronic diseases. Acute conditions include chemical injuries, Stevens-Johnson syndrome, and ocular surface burns. Chronic diseases encompass dry eye, chronic cicatrizing conjunctivitis, limbal stem cell deficiency (LSCD), vernal keratoconjunctivitis (VKC), Sjögren's syndrome, peripheral ulcerative keratitis, ocular mucous membrane pemphigoid (MMP), and other end-stage diseases [1].

These debilitating end-stage corneal surface diseases significantly impair both vision and quality of life. Corneal perforations resulting from these severe ocular surface disorders are a significant cause of blindness, particularly in developing countries. Effective and timely management of these perforations is crucial to maintaining structural integrity and preventing complications such as secondary glaucoma, endophthalmitis, and phthisis bulbi. Depending on size, location, co-existing ocular health, and the disease process, various surgical procedures are available for managing perforations [2]. Considering the above, treatment must be individualized and adapted, as a one-size-fits-all approach is ineffective [3]. Therefore, managing corneal perforations in these conditions necessitates a multipronged approach, addressing the ocular surface, the corneal defect, and the underlying ocular or systemic disease [4].

This case series, along with a review of the literature, highlights the chronic and end-stage diseases in which the ocular surface is compromised, and chronic inflammation occurs. These eyes are prone to repeated breaches in ocular integrity, requiring immediate intervention to prevent associated complications.

## Case presentation

Case 1: tissue adhesive with bandage contact lens

**Figure 1:**
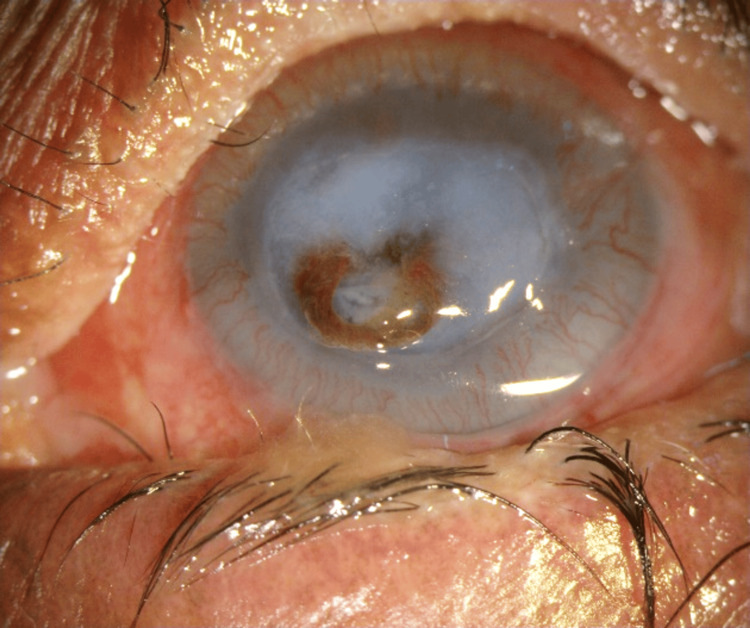
Inferior corneal melt with a pinpoint leak and iris prolapse

A 65-year-old woman with a history of Stevens-Johnson Syndrome presented with severe photophobia and foreign body sensation in both eyes. Examination revealed bilateral lid margin keratinization, which was more severe in the right eye, and cicatricial entropion of the upper eyelid in the right eye. She underwent right eye entropion correction with mucous membrane and auricular cartilage grafting but kept developing conjunctivitis. In the course of follow-up, she developed an inferior large corneal melt with iris prolapse and a pinpoint leak (Figure 1).

She underwent tissue adhesive with a bandage contact lens (BCL) and amniotic membrane, followed by anterior chamber reformation. She was also maintained on systemic immunosuppression to manage the active inflammation.

**Figure 2:**
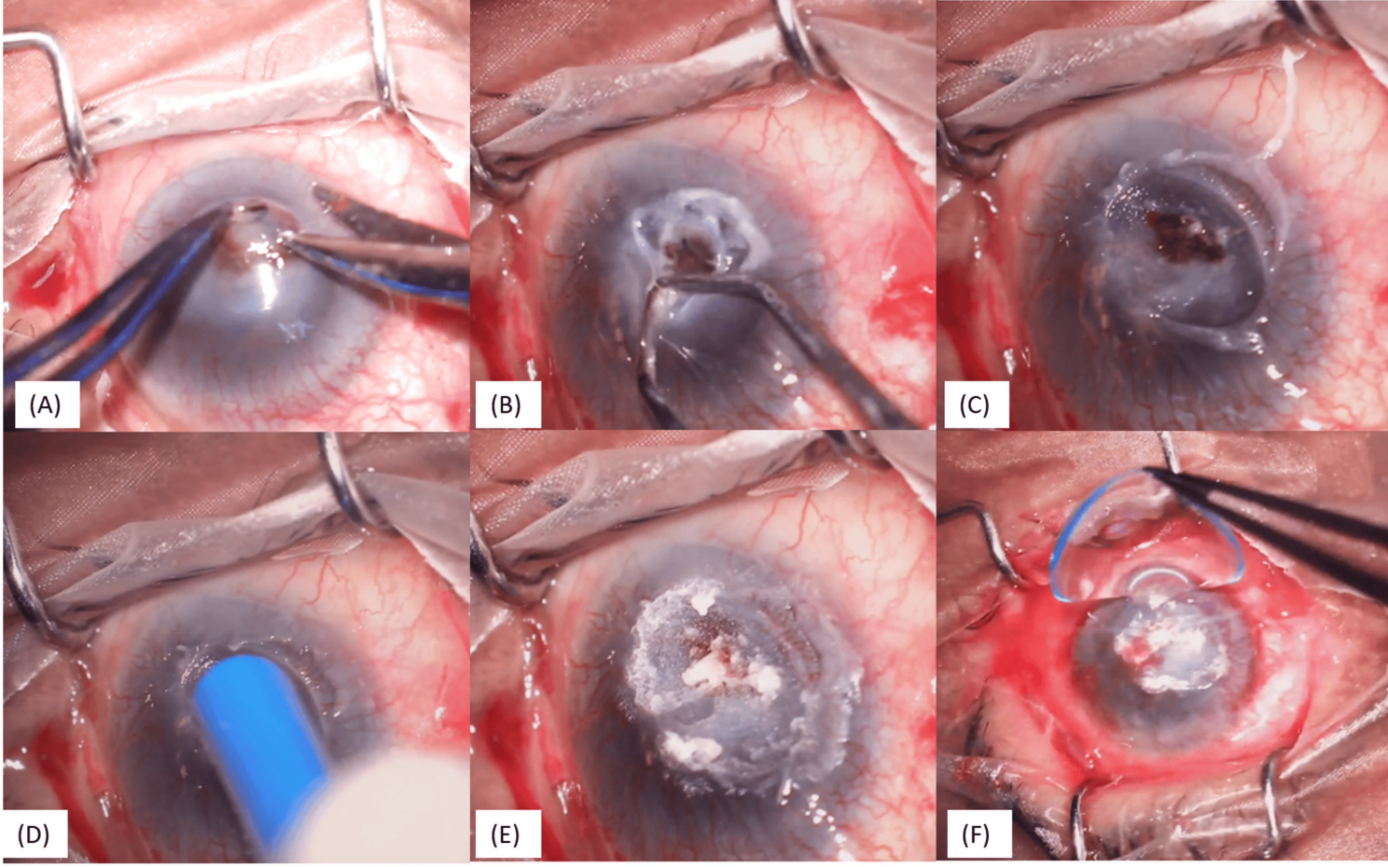
Steps of cyanoacrylate tissue adhesive (CTA) with bandage contact lens (BCL) (A-C): Debridement of loose epithelium with forceps and cauterization if needed; (D-E): application of CTA with the back of merocele sponge on the de-epithelized cornea; (F): BCL placed

The loose epithelium was debrided during surgery, 360-degree cautery was performed around the defect, tissue adhesive was applied over the leak area, and the anterior chamber was reformed with a balanced salt solution (Figures 2A-2F). Fibrin glue was then applied on the periphery of the cyanoacrylate tissue adhesive (CTA), and a small piece of amniotic membrane was placed to cover the CTA and the entire area of the debrided epithelium. After that, a bandage contact lens was placed.

**Figure 3:**
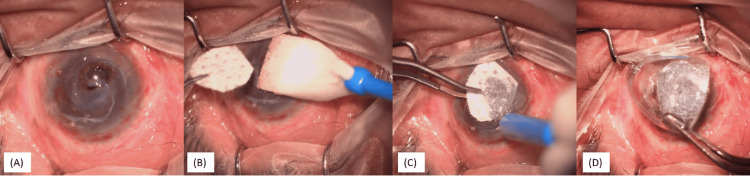
Steps of drape tissue adhesive with bandage contact lens (BCL) (A): stromal bed prepared; (B, C): 3*3 mm sterile drape applied with tissue adhesive; (D) BCL placed

Postoperatively, she was stable for six weeks. At six weeks, she presented with displaced tissue adhesive, a flat anterior chamber, and severe corneal thinning. Considering that the tissue adhesive did not provide adequate support the second time, she was planned for a repeat procedure. A 3*3 mm sterile plastic drape was placed over the defect, after which the tissue adhesive was applied (Figures 3A-3C).

**Figure 4:**
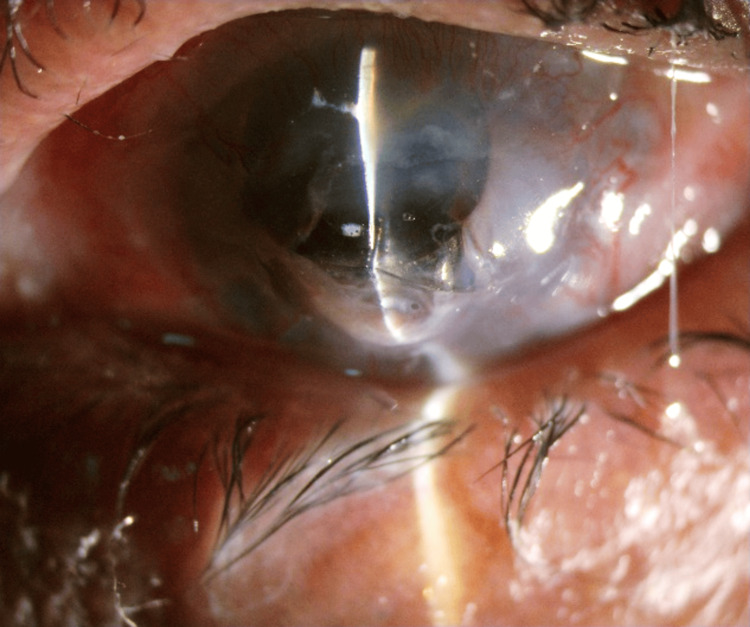
One-month post-drape tissue adhesive with bandage contact lens (BCL), inferior corneal melt with intraocular lens (IOL) extrusion noted

She was stable for one month, after which she developed repeat graft melt for which she underwent intraocular lens (IOL) explantation, anterior vitrectomy, and a corneal patch graft (Figure 4).

This case highlights the limitations of cyanoacrylate glue in managing larger than pinpoint perforations in the presence of active surface inflammation. In these disorders, the tear film is inadequate so the BCL does not stay well on the ocular surface and gets dislodged. Also, during the healing process, CTA attracts new vessels, which can be detrimental to these disorders, and hence a more bio-integrable tissue is desirable.

Case 2: Tenon's patch graft

A 49-year-old man presented to us with a complaint of severe dry eye. He had undergone a bone marrow transplant for chronic myeloid leukemia (CML) in December 2020 elsewhere and was diagnosed with graft versus host disease (GVHD). At presentation, visual acuity in both eyes was 6/12 (logMAR 0.30). He had severe photophobia at the time of presentation. On examination, he had bilateral punctate epithelial erosions with paracentral anterior stromal scarring in both eyes. The patient was kept on a maintenance dose of topical steroids and lubricants.

**Figure 5:**
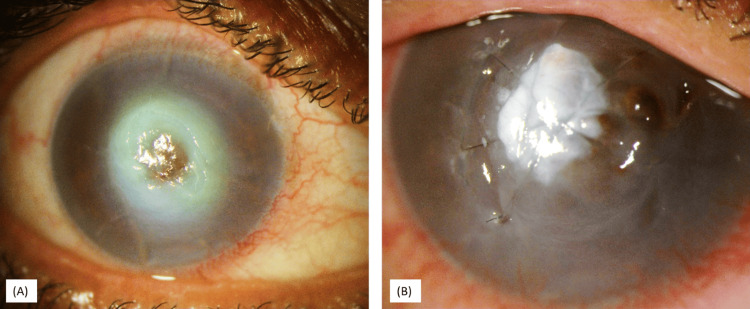
(A): Central 3 mm perforation with surrounding 7 mm thinning; (B): One month post Tenon's patch graft

In 2023, the patient developed a central persistent epithelial defect in the right eye, for which he underwent amniotic membrane grafting and was started on topical and oral immunosuppressants. The epithelial defect healed; however, the patient developed repeated episodes of recurrent epithelial defect in consecutive months, culminating in a central 3 mm corneal perforation with surrounding 7 mm thinning (Figure 5A). Since the patient had multiple episodes of epitheliopathy, severe LSCD, and corneal thinning, a Tenon's patch graft (TPG) with anterior chamber formation was planned.

Tenon’s layer was obtained from the inferonasal quadrant by carefully separating it from the overlying conjunctiva. Following the removal of the surrounding epithelial tissue, the Tenon’s layer was positioned over the defect area using fibrin glue. To enhance stability, sutures were applied to secure the layer in place. The anterior chamber was subsequently reformed with saline. BCL was placed. The patient is doing well at six months post-surgery (Figure 5B).

Thus, this case underscores the significance of Tenon’s patch grafting (TPG) in managing severe ocular surface diseases with larger perforations, approximately three millimeters in size, where penetrating keratoplasty or patch grafts may not be a viable option.

Case 3: surface mucous membrane grafting

A 55-year-old woman with a known history of Stevens-Johnson Syndrome (SJS) was referred to our hospital. The patient developed SJS in 2022 after the intake of non-steroidal anti-inflammatory drugs (NSAIDs) and was referred to our center in 2024. The patient had undergone bilateral amniotic membrane grafting with lateral tarsorrhaphy in both eyes during the acute phase of the disease. The patient at the presentation had a visual acuity of perception of light with accurate projection of rays in both eyes with 360-degree symblepharon formation and total LSCD. The left eye had a 4 mm sealed epithelized perforation with iris prolapse in the inferonasal cornea paracentrally. Lid margin keratinization, along with severe corneal thinning, was seen in the right eye. A staged procedure was thus planned for the right eye in order to stabilize the ocular surface and make an effort to restore ambulatory vision. She was thus planned for and underwent right eye surface mucous membrane grafting. No intervention was planned for the left eye, as the area was well-epithelized and quiet with an irregular anterior segment.

**Figure 6:**
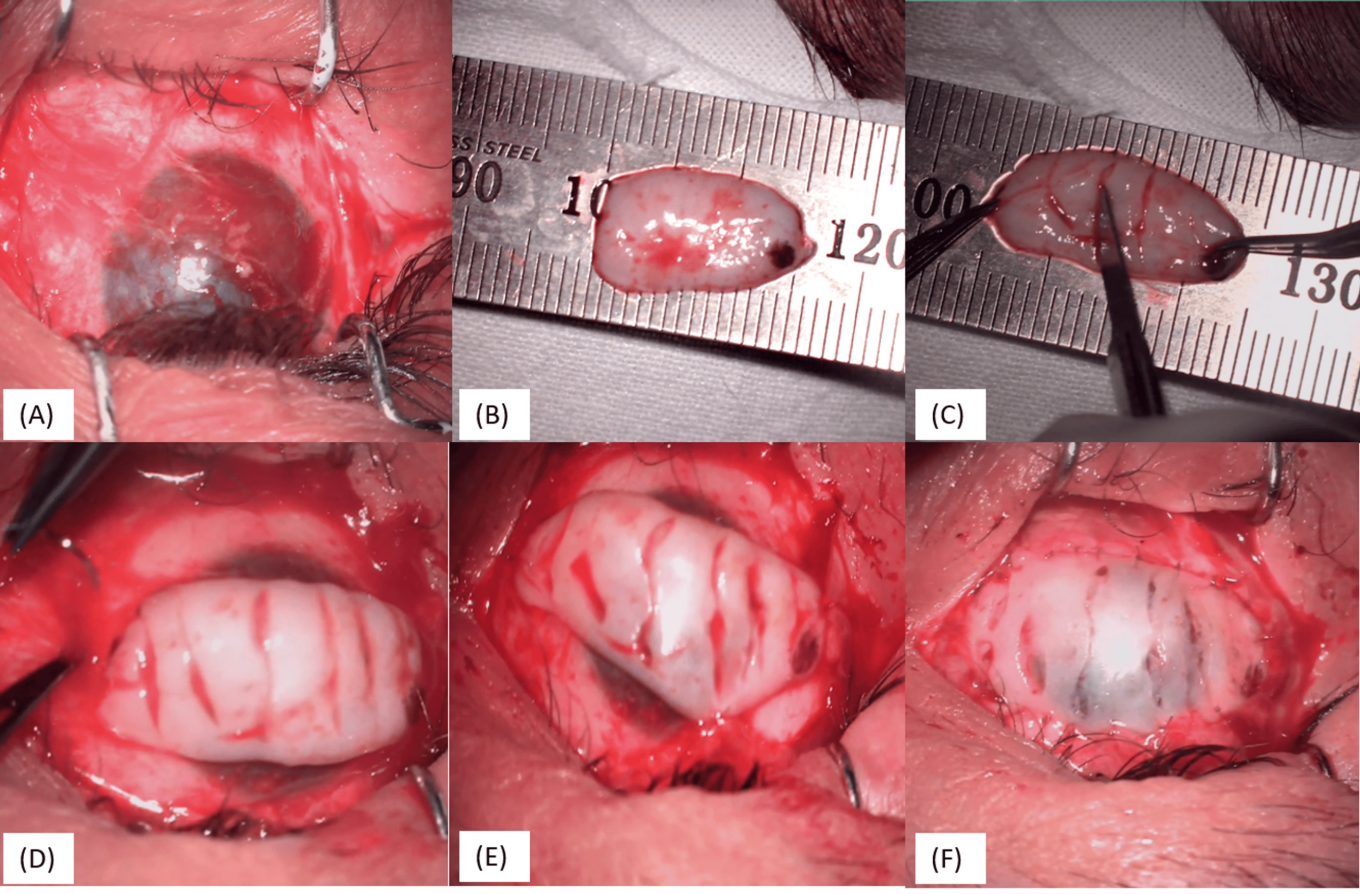
Surgical steps of surface mucous membrane grafting (A): Symblepharon release done, pannus over the central cornea was left in situ; (B) Harvested oral mucosa from the lower lip; (C) multiple partial-thickness vertical incisions made to increase the surface area of the mucosal graft; (D-F): Graft secured to the host tenons with 7-0 vicryl sutures at the four recti and conjunctiva to ensure proper vascular supply of the graft

In this procedure, lateral cantholysis was performed, followed by symblepharon release and pannus dissection over the bulbar conjunctiva. Pannus over the central cornea was left in situ to provide a protective fibrovascular layer, adding volume and structural support to the thinned cornea, thereby reducing the risk of further perforation. Tenon's tissue was released from the fornix. Following this, oral mucosa was harvested from the lower lip. The donor site was sutured with 6-0 vicryl sutures. Oral mucosa was placed over the ocular surface, and multiple partial thickness vertical incisions were made to increase the surface area of the mucosal graft. The graft was secured to Tenon’s capsule and the scleral insertion sites of the four recti muscles using 7-0 vicryl sutures to ensure proper vascular supply (Figures 6A-6F). Temporary suture tarsorrhaphy was then done.

**Figure 7:**
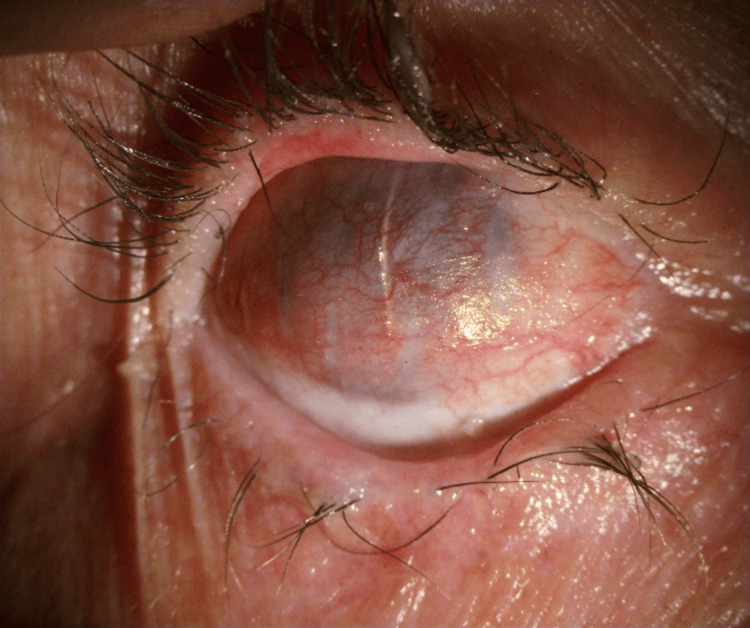
Three months post surface mucous membrane grafting.; ocular surface stable

The patient is doing well three months post-surgery with a stable vascularised ocular surface (Figure 7).

The second staged procedure would include planning the patient for a keratoprosthesis to restore the patient's vision.

Case 4: scleral patch grafts

A 33-year-old man who had suffered from a chemical injury in 2010 in both eyes had undergone penetrating keratoplasty in the right eye and Boston keratoprosthesis with amniotic membrane grafting and tarsorrhaphy in the left eye. In 2019, the visual acuity in the right eye was counting finger (CF) at 1 meter, owing to the failed graft. His vision in the left eye had fallen from 6/12 (logMAR 0.30) to hand movement (HM) vision owing to glaucomatous cupping in the left eye.

For visual rehabilitation, the patient was planned for and underwent a repeat optical penetrating keratoplasty with allogenic simple limbal epithelial transplantation (alloSLET) and tarsorrhaphy in the right eye in 2019. He maintained a visual acuity of 6/18 (logMAR 0.50) for around five years (December 2023). He had two episodes of epithelial defect post keratoplasty, which required amniotic membrane grafting owing to severe LSCD.

After around five years (January 2024), he developed a small paracentral perforation at the graft host junction with flat AC, for which he underwent CTA with BCL. From January until April, the patient underwent repeat CTA with BCL twice, as repeated perforation was noted at the same site. A tenon's patch graft was performed in May 2024, followed by a sclerocorneal patch graft with amniotic membrane grafting in June 2024.

**Figure 8:**
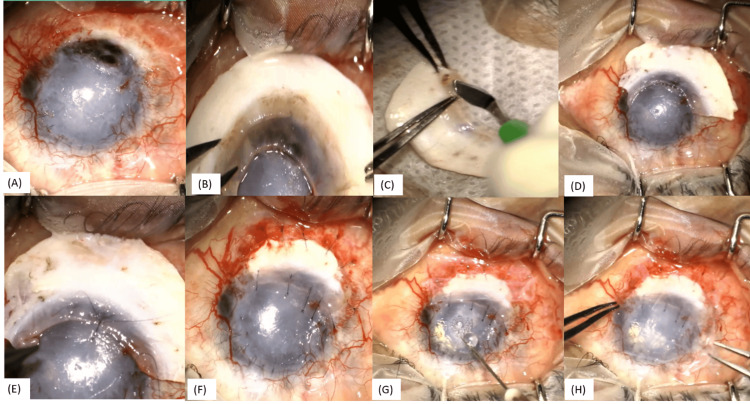
Surgical steps of the scleral patch graft (A): 4*3mm paracentral perforation at the graft host junction; (B-D): measurement and preparation of the scleral rim of the required size; (E, F): Rim sutured to the host sclera and cornea with 10-0 nylon sutures; (G, H): Amniotic membrane placed over the entire ocular surface with fibrin glue

In this procedure, the scleral rim of the required size was measured, placed over the defect, and then sutured to the host sclera and cornea with 10-0 nylon sutures. The amniotic membrane was then placed over the entire ocular surface with fibrin glue (Figures 8A-8H).

Case 5: conjunctival flaps/Gunderson flaps

A 42-year-old man with a history of chemical injury with caustic soda in both eyes in December 2020 presented to us in 2022 with complaints of pain and diminished vision in both eyes. He had undergone bilateral multiple amniotic membrane transplantations along with tenonplasty, followed by right-eye optical penetrating keratoplasty with extracapsular cataract extraction (ECCE) and IOL implantation with Allo-SLET and tarsorrhaphy surgery in 2021 and left-eye ECCE+ IOL in 2022, elsewhere. The patient was already on topical immunosuppressants and systemic immunomodulators.

At presentation, visual acuity in both eyes was counting fingers close to the face. The right eye had a central 7 mm corneal melt, and the left showed a scarred and vascularised cornea. To provide ambulatory vision to the patient, left eye keratoprosthesis surgery with amniotic membrane transplantation was performed in November 2011. Central permanent tarsorrhaphy was done in the right eye. Visual acuity post keratoprosthesis surgery was 2/60 in the left eye, and the ocular surface eventually stabilized in the right eye.

**Figure 9:**
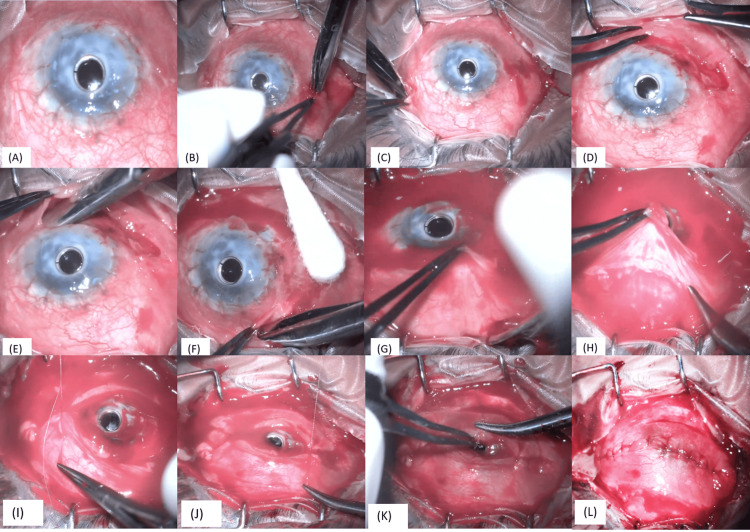
Surgical steps of a conjunctival flap (A) Pericylindrical melt in keratoprosthesis; (B, C) Conjunctival peritomy done nasally and temporally; (D-F): Conjunctiva mobilized 360 degrees; (G, H): Superior and inferior forniceal conjunctiva separated from the tenon capsule; (I-L): Conjunctiva sutured over the keratoprosthesis with 6-0 vicryl sutures

In January 2022, left eye peri-cylindrical melt was noted, and the patient underwent a Gunderson conjunctival flap with tarsorrhaphy for the same. Intraoperatively, conjunctival peritomy was done nasally and temporally, and conjunctiva was mobilized 360 degrees. Superior and inferior forniceal conjunctiva was separated from the tenon capsule. The conjunctiva was sutured over the keratoprosthesis with 6-0 vicryl sutures (Figures 9A-9L).

**Figure 10:**
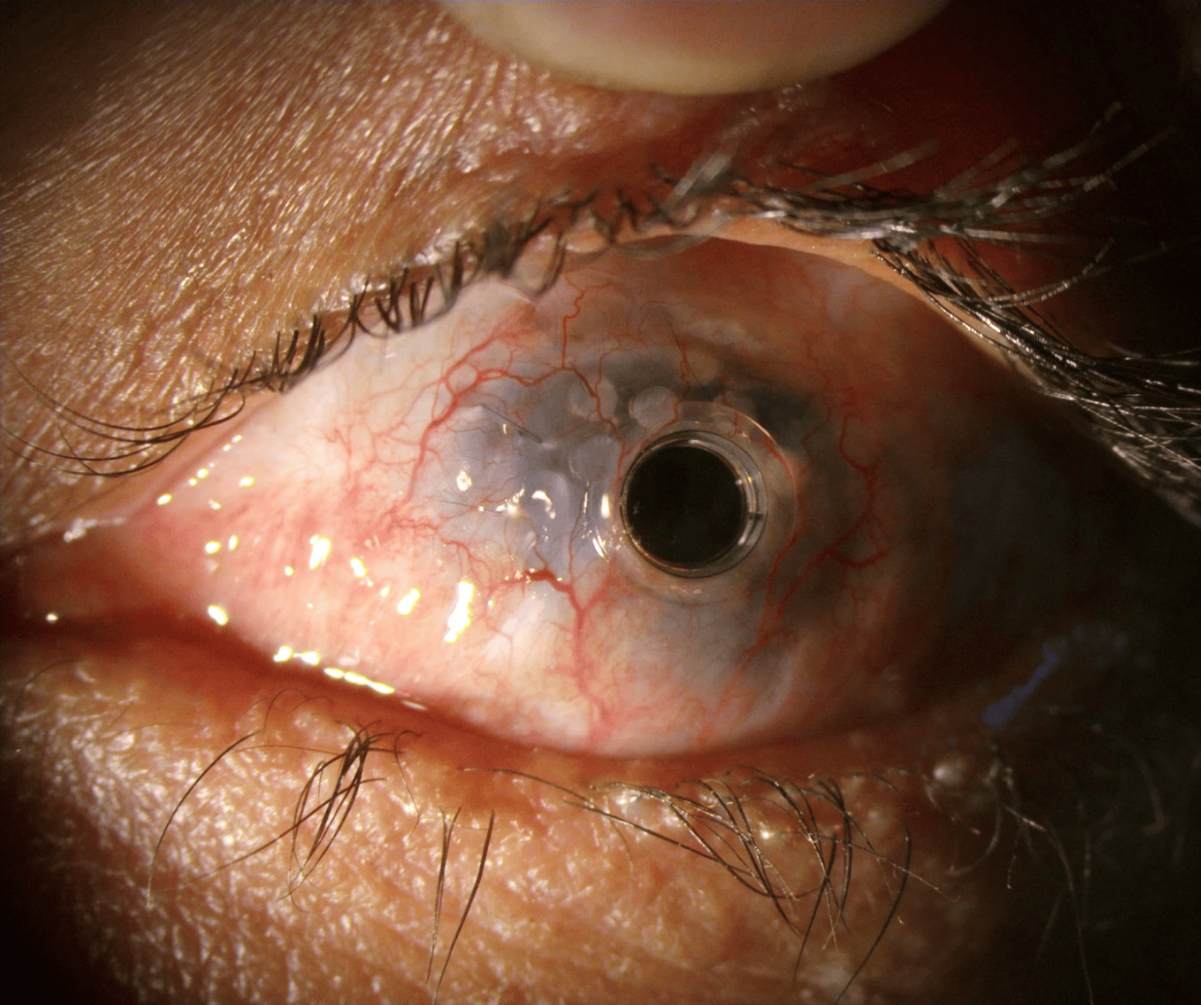
Two years post conjunctival flap for pericylindrical melt; stable ocular surface

Paramedian tarsorrhaphy was done. The patient's last follow-up was in March 2024, and his best-corrected visual acuity was 6/18 (logMAR 0.50) with a stable ocular surface (Figure 10).

## Discussion

Corneal melts and perforations in chronic ocular surface disorders arise due to a complex interplay of factors disrupting the ocular surface integrity. Three fundamental elements - a stable preocular tear film, an intact epithelial barrier, and normal adnexal anatomy - are essential for maintaining corneal health. Disruptions in any of these components can lead to non-healing epithelial defects, which, in severe cases, progress to stromal thinning and perforation [5]. Persistent inflammation, often seen in Stevens-Johnson Syndrome (SJS), graft-versus-host disease (GVHD), and chemical injuries, plays a central role in corneal melting and perforation by exacerbating ocular surface instability.

In SJS, a type IV hypersensitivity reaction triggers extensive keratinocyte apoptosis. The immune response, mediated by CD8 cytotoxic cells and natural killer cells via granulysin release, leads to mitochondrial damage and apoptosis, ultimately causing severe adnexal and ocular surface changes [6]. These changes create a cycle of inflammation and microtrauma, predisposing the cornea to persistent epithelial defects and perforation. Similarly, in chronic ocular GVHD, corneal perforations result from sustained inflammatory infiltration and a dry eye microenvironment. Matrix metalloproteinase-9 (MMP-9) has been implicated in corneal melting in GVHD, with studies demonstrating its presence in the perforation margins, further contributing to stromal degradation [7]. Corneal perforations due to chemical injuries follow a similar pathophysiology, with enzymatic activity and chronic inflammation leading to corneal instability and progressive thinning [8].

Clinically, corneal perforations manifest as sudden visual deterioration, pain, and increased tearing. While many perforations remain sterile, a microbiological evaluation is necessary to rule out secondary infections. A shallow or flat anterior chamber, positive Seidel’s test, uveal prolapse, and hypotony are common signs, necessitating immediate intervention [9]. The choice of management depends on the size and location of the perforation. Tissue adhesives, such as cyanoacrylate glue, are effective for small perforations. At the same time, larger defects may require surgical approaches, including conjunctival pedicle flaps, Tenon’s patch grafts, corneal patch grafts, or penetrating keratoplasty [10].

Cyanoacrylate tissue adhesive (CTA) is widely used for sealing perforations up to 2 mm, acting as a barrier to stromal lysis, supporting re-epithelialization, and offering bacteriostatic properties [11]. However, its long-term efficacy varies (29%-86% success), often requiring secondary surgical interventions [9].

For larger perforations, Tenon’s patch grafting (TPG) provides a vascularized, autologous tissue source that supports healing, reduces inflammation, and avoids immune reactions. Its affordability and availability make it a preferred emergency graft [12]. Patel et al. reported epithelial healing within three months in auro-keratoprosthesis cases [13], while Anitha et al. expanded its use with Gore-Tex for larger perforations [14].

Oral mucosa grafting is effective for severe ocular surface disorders like SJS, offering biological similarity to conjunctiva and minimizing immune rejection [15]. It has shown success in treating mucin deficiency syndromes and preparing the ocular surface for keratoprosthesis [16].

Scleral patch grafting with tissue adhesives is a promising alternative to corneal patch grafts (CPG) for moderate-sized perforations, providing tensile strength, availability, and reduced immune rejection risk [17].

When conventional methods fail, conjunctival flaps act as a last resort, covering perforations, reducing tear film exposure, and promoting healing. With up to 95% efficacy, they remain reliable but have suboptimal cosmetic and functional outcomes [18].

Table 1 briefly discusses the surgical and non-surgical techniques for managing corneal perforations.

**Table 1:** Comparison of surgical and non-surgical techniques for managing corneal perforations

Treatment Option	Description	Advantages	Limitations	Success Rate
Cyanoacrylate Tissue Adhesive (CTA)	Seals perforations up to 2 mm, acts as a barrier, provides a scaffold for re-epithelialization, and has bacteriostatic properties	Quick application, prevents further stromal lysis, supports healing	Variable long-term efficacy, may require secondary surgery	29%–86%
Tenon’s Patch Grafting (TPG)	Uses vascularized autologous Tenon’s capsule tissue for epithelial migration, inflammation reduction, and wound healing	Autologous (no immune rejection), inexpensive, readily available, effective for emergency cases	Limited data on long-term outcomes	Demonstrated epithelial healing within 3 months
Oral Mucosa Grafting	Utilizes oral mucosa as a substitute for conjunctiva in patients with severe ocular surface disorders (e.g., SJS)	Biologically similar to conjunctiva, reduces immune rejection, accessible	Limited cosmetic outcomes, requires specialized surgical skills	Successful in mucin deficiency syndromes and ocular surface reconstruction
Scleral Patch Grafting	Uses scleral tissue combined with adhesives for moderate-sized perforations in immune-mediated conditions	High tensile strength, reduces immune rejection risk, durable	Limited comparative studies with other methods	Promising alternative to corneal patch grafts
Conjunctival Flaps	Covers perforation, reduces exposure to inflammatory mediators, and promotes healing	High efficacy (up to 95%), last-resort option when other treatments fail	Poor cosmetic and functional outcomes	Up to 95% efficacy

Recent advancements in biomaterials offer promising alternatives for managing corneal perforations. Cell-free biosynthetic hydrogels and collagen-like peptides (CLP) have been explored, with Griffith et al. demonstrating the efficacy of a CLP-polyethylene glycol (PEG)-fibrinogen mixture cross-linked with 4-(4,6-Dimethoxy-1,3,5-triazin-2-yl)-4-methylmorpholinium chloride (DMTMM) in sealing full-thickness perforations in animal models. Human recombinant collagen hydrogels combined with mesenchymal stem cells are also being investigated for stromal tissue regeneration, offering benefits such as defect filling, structural support, and epithelial integration [19]. Similarly, Kuragel, a biodegradable hydrogel composed of functionalized gelatin and hyaluronic acid, is under evaluation for corneal regeneration [20].

Despite these advances, patients remain at risk for delayed epithelial healing and recurrent perforation. To prevent complications, topical and systemic therapies are essential for promoting epithelialization and preventing stromal melt. Visual rehabilitation may involve spectacles, scleral contact lenses, or cataract surgery, while keratoplasty should be reserved for refractory cases. If lens replacement fails to restore vision, keratoprosthesis may be considered.

## Conclusions

The management of corneal perforations depends on the lesion's size, shape, location, and underlying cause. Smaller perforations may be effectively treated with tissue adhesives, Tenon’s patch grafting, or amniotic membrane transplantation. These approaches help stabilize the cornea and promote healing without more invasive procedures. However, larger perforations often require urgent keratoplasty to restore the cornea's structural integrity and prevent further complications.

Recent advancements in corneal surgery focus on lamellar corneal tissue, which involves transplanting only the affected layers of the cornea rather than a full-thickness graft. This technique aims to reduce the risk of rejection and improve clinical outcomes by preserving more of the patient's healthy corneal tissue. Ongoing research and development in this area continue to enhance the effectiveness and safety of treatments for corneal perforations.
